# Multicystic encephalomalacia and gastrointestinal injury following single fetal death in first trimester and subsequent fetofetal transfusion syndrome in a monochorionic triplet pregnancy: a case report

**DOI:** 10.1186/s12884-019-2459-4

**Published:** 2019-08-27

**Authors:** Po Lam So, Ka Wah Li, Tsz Wai Yeung, Wai Kuen Sin

**Affiliations:** 1 Department of Obstetrics and Gynecology, Tuen Mun Hospital, 23 Tsing Chung Koon Road, Tuen Mun, Hong Kong, Special Administrative Region of China; 2 Department of Paediatrics and Adolescent Medicine, Tuen Mun Hospital, 23 Tsing Chung Koon Road, Tuen Mun, Hong Kong, Special Administrative Region of China; 3 Department of Radiology, Tuen Mun Hospital, 23 Tsing Chung Koon Road, Tuen Mun, Hong Kong, Special Administrative Region of China

**Keywords:** Monochorionic triplet pregnancy, Single fetal death, Fetofetal transfusion syndrome, Multicystic encephalomalacia, Gastrointestinal injury

## Abstract

**Background:**

Monochorionic multifetal pregnancies are at increased risk of adverse perinatal outcome because of placental vascular anastomoses. We present a case of multicystic encephalomalacia and gastrointestinal injury in two surviving fetuses following single fetal death in first trimester and subsequent fetofetal transfusion syndrome in a monochorionic triplet pregnancy.

**Case presentation:**

A 31-year-old nulliparous woman had a spontaneous monochorionic triamniotic triplet pregnancy. Three live fetuses with single placenta were seen at 8-week ultrasound scan. One fetus demised at 11 weeks and 3 days of gestation. Dilated echogenic bowel and ascites were found in one surviving fetus at 23 weeks of gestation. At 28 weeks of gestation, the pregnancy was complicated by fetofetal transfusion syndrome in which discordant amniotic fluid volumes were found. Two days later, emergency Caesarean section was performed because of worsening of fetal Doppler and biophysical profile. One baby was found to have jejunal atresia requiring surgery at 4 days old. He had periventricular leukomalacia and intracranial haemorrhage, but subsequent normal neurological development. Another baby had gastric perforation requiring surgery at 2 days old. He was confirmed to have multicystic encephalomalacia by cranial ultrasound and magnetic resonance imaging. He suffered from developmental delay, epilepsy and cerebral palsy.

**Conclusion:**

This case alerts the obstetricians the possible hypoxic-ischemic injury to the survivors of monochorionic triplet pregnancy after the co-triplet death in the first trimester and fetofetal transfusion syndrome. Antenatal assessment and postnatal follow-up are important for these high-risk multiple pregnancies.

## Background

Fetal brain injuries are reported in the survivors of twin pregnancies complicated by demise of the co-twin and fetofetal transfusion syndrome [1, 2]. However, the literature concerning the neurological outcomes of survivors after the co-triplet death and fetofetal transfusion syndrome is limited. Triplet and higher-order births accounted for 101.4 per 100,000 births in the United States in 2016 [3]. Similar to twin pregnancies, chorionicity is an important determinant of fetal outcomes in triplet pregnancies. Observational studies show that monochorionic or dichorionic triplet pregnancies carry significantly higher risk of perinatal loss compared with trichorionic triplet pregnancies [4, 5]. The underlying cause is shared fetoplacental circulation with specific pregnancy complications including fetofetal transfusion syndrome, selective fetal growth restriction and co-triplet death. We report a case of neurologic and gastrointestinal complications in two surviving fetuses following single fetal death in first trimester and subsequent fetofetal transfusion syndrome in a monochorionic triplet pregnancy.

## Case presentation

**Fig. 1:**
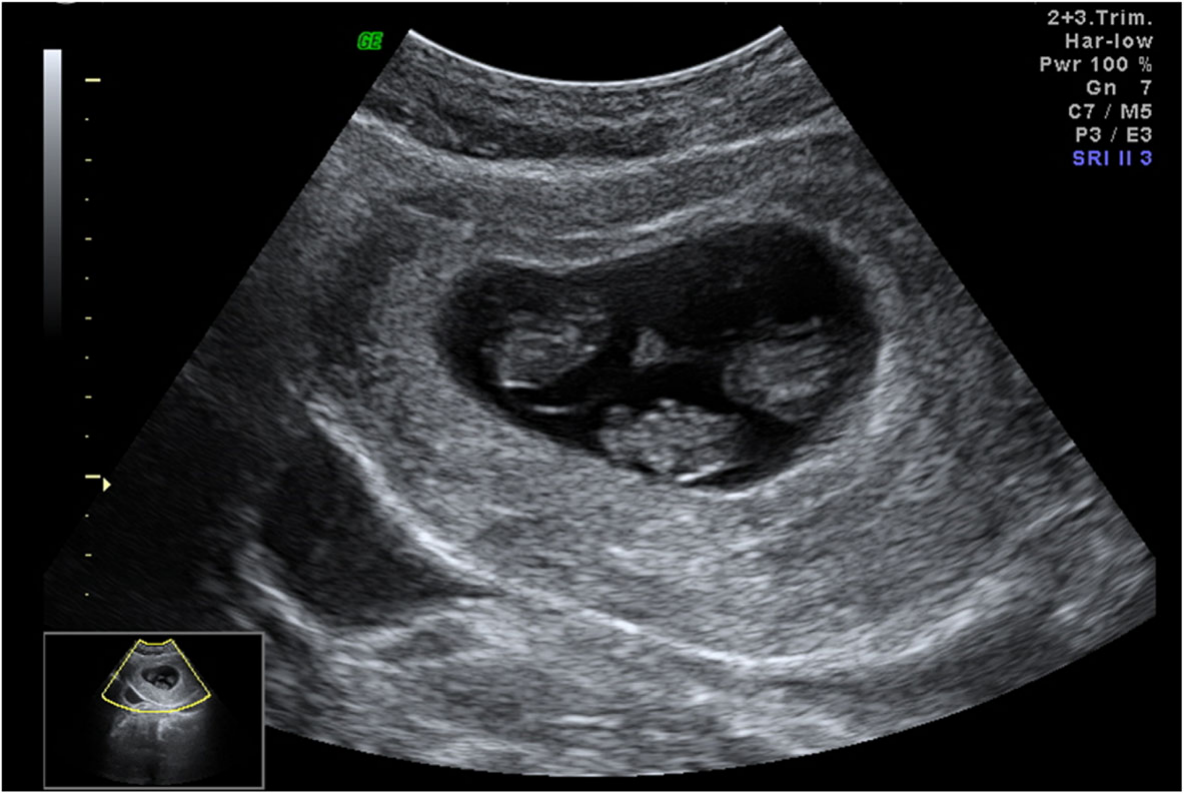
Prenatal transabdominal ultrasound at 8 weeks of gestation demonstrated three viable fetuses with one shared placenta and three amniotic cavities

A healthy 31-year-old nulliparous Chinese woman had a spontaneous monochorionic triamniotic triplet pregnancy. Three live fetuses (fetus 1, fetus 2, fetus 3) were seen at 8-week and 9-week ultrasound scans with crown-rump length measurements appropriate for gestational age which was calculated by last menstrual period. The scan showed a single placental mass, two intertwin membranes with T-sign and three amniotic sacs (Fig. 1). At 11 weeks and 3 days of gestation, an ultrasound examination found fetus 1 measured 11 weeks and 2 days without cardiac activity. The surviving fetus 2 and fetus 3 measured 11 weeks and 2 days and 11 weeks and 4 days respectively. Fetal growth, liquor volume and umbilical artery Dopplers were assessed every 2 weeks. At 19 weeks’ gestation, the dead fetus was not visualized and anomaly scan including the central nervous system for the two remaining fetuses was normal. There was no ultrasound feature of fetofetal transfusion syndrome. However, at 23 weeks of gestation, ultrasound evaluation revealed the fetus 2 with dilated echogenic bowel (2 cm) and ascites. Fetal Doppler and growth were normal for both fetuses. The patient was informed that the findings were likely a result of underlying bowel abnormality. She declined invasive prenatal diagnostic test because of approaching the 24-week abortion limit. She chose to carry on the pregnancy.

At 28 weeks of gestation, the pregnancy was complicated by fetofetal transfusion syndrome. There was oligohydramnios (a maximum amniotic pool depth of 2 cm) in the fetus 2 (donor) and polyhydramnios (a maximum amniotic pool depth of 12 cm) in the fetus 3 (recipient). Amnioreduction was not performed because the mother did not have significant distending symptom. In view of possible rapid deterioration, a course of corticosteroid was administered for fetal lung maturation. Two days later, she complained of decreased fetal movement of one fetus. Ultrasound examinations revealed the fetus 2 with anhydramnios, invisible bladder and biophysical profile of zero. The fetus 3 had increased cardiothoracic ratio, significant tricuspid regurgitation and reverse flow in ductus venosus. The patient opted for immediate delivery. Two male babies were delivered by emergency Caesarean section. The first baby which was fetus 2 weighed 915 g (10th centile) and had Apgar scores of 5 and 9 at 1 and 5 min, respectively. The second baby which was fetus 3 weighed 980 g (25th centile) and had Apgar scores of 6 and 8 at 1 and 5 min respectively.

Both babies were transferred to the neonatal intensive care unit for ventilation and received surfactant for respiratory distress syndrome. The hemoglobin levels of the first and second babies were 15.8 and 18.0 g/dL, respectively (a difference of 2.2 g/dL). An abdominal X-ray for bowel evaluation of the first baby showed a triple bubble sign, suggestive of small bowel obstruction. Laparotomy was done at 4 days old. The operative findings were jejunal atresia and small bowel volvulus. Small bowel resection was performed. The second baby was noted to have abdominal distension at 2 days old. An abdominal X-ray showed pneumoperitoneum and dilated bowel loops. Laparotomy was done and revealed gastric perforation. Repair of stomach and gastrostomy were performed. Both babies had uncomplicated operations and recovery of bowel function.

The brain ultrasound at 22 days old of the first baby showed bilateral periventricular hyperechogenicity and a grade 1 germinal matrix haemorrhage at right caudothalamic groove. On last follow-up at age 5 years, the child had normal growth and development.

**Fig. 2:**
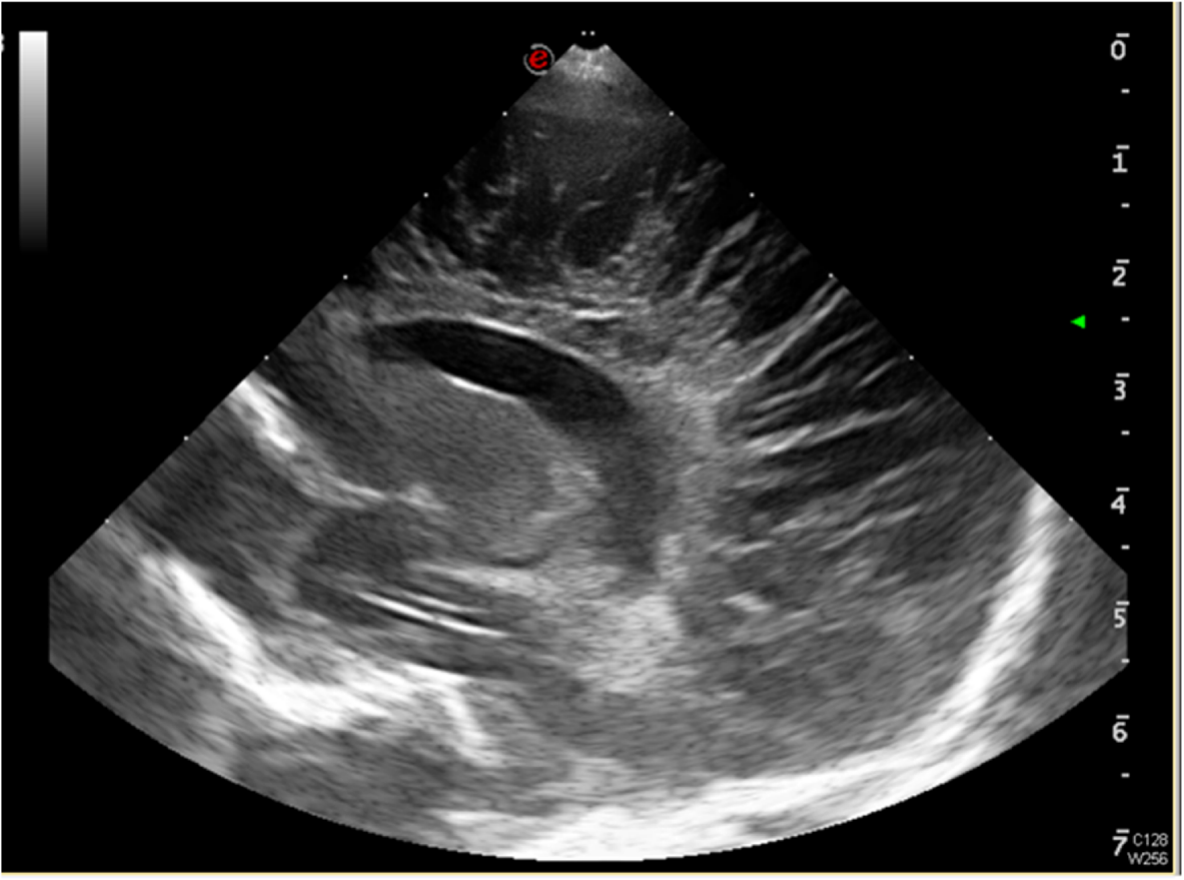
Postnatal transfontanelar ultrasound of second baby at twenty-second day of life showing multiple cystic cavities in bilateral periventricular regions

**Fig. 3:**
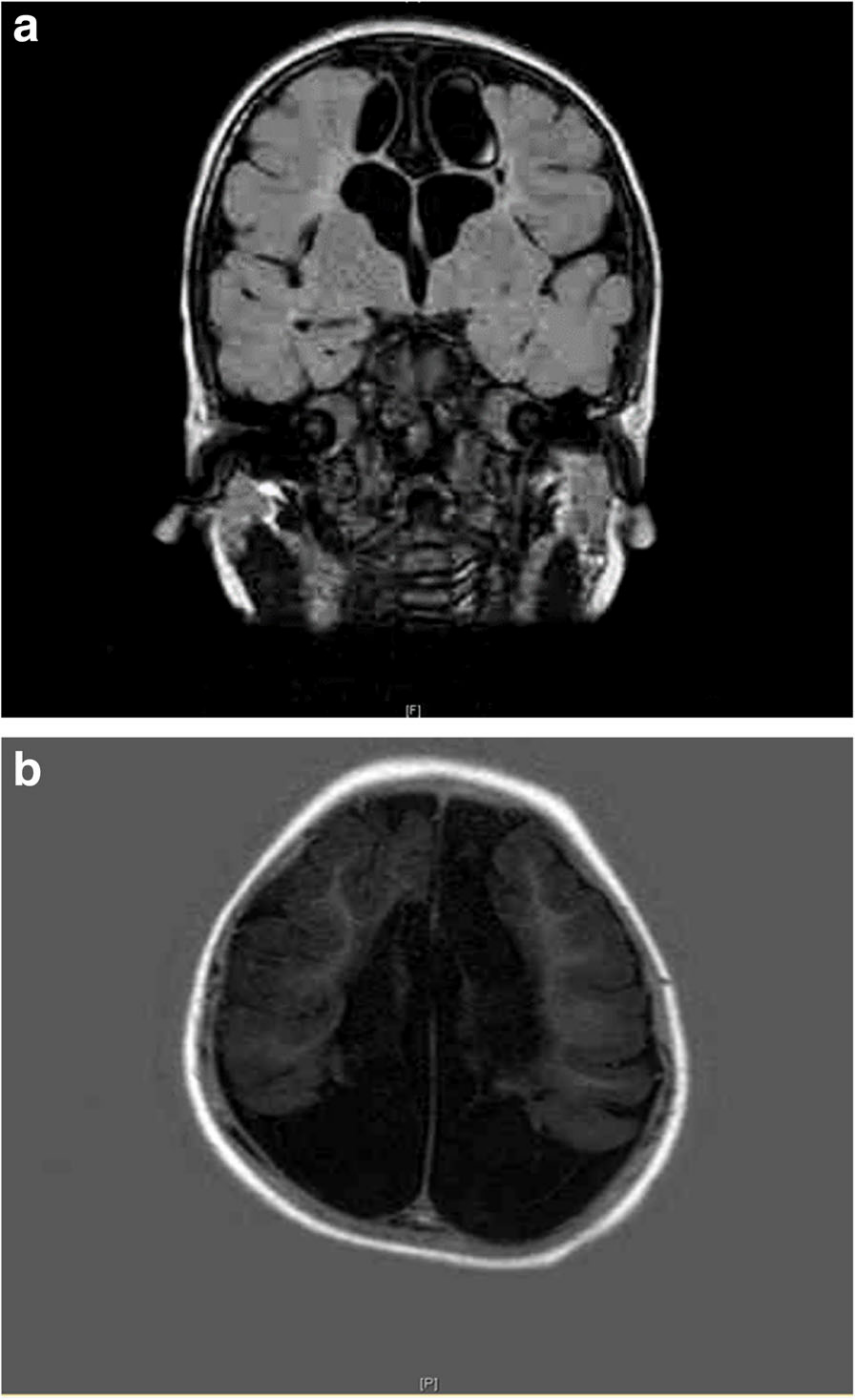
Postnatal magnetic resonance imaging of the brain (**a**) coronal T2- weighed FLAIR image and (**b**) axial T1- weighed image of second baby at 1.5 years of age showing microcephaly, extensive loss of supratentorial brain parenchyma replaced by multiple cystic spaces, more severely at bilateral peritrigonal and parafalcial regions, with ex-vacuo dilatation of bilateral ventricles, also severe thinning of the corpus callosum

**Fig. 4:**
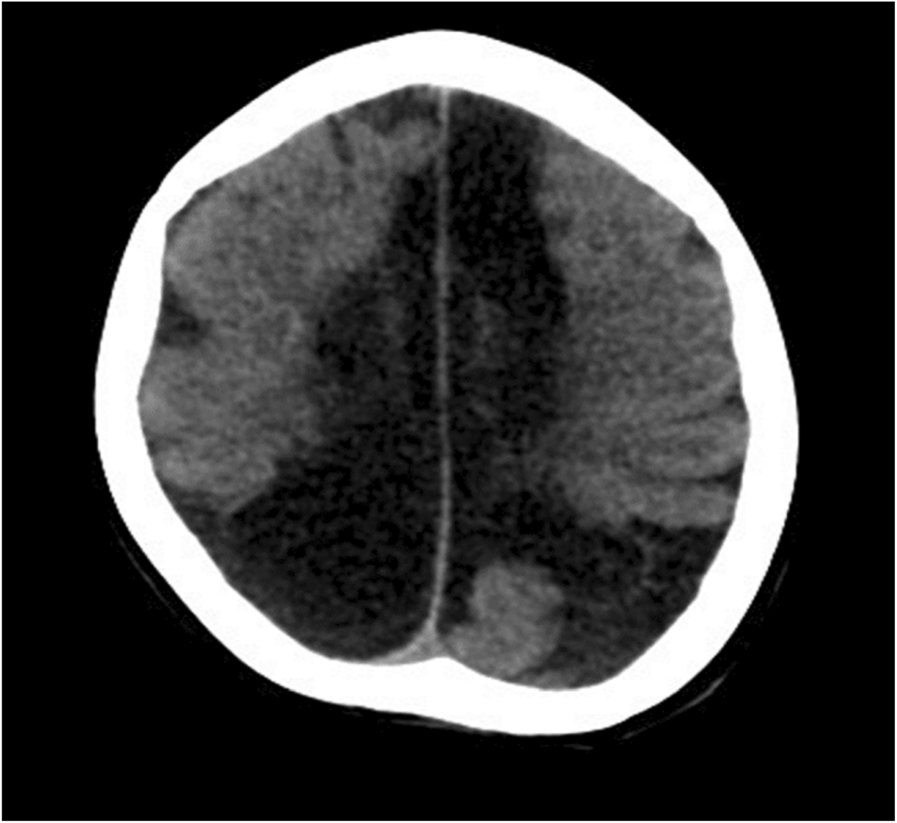
Postnatal head computed tomography scan of second baby at 6 years old showing hypodense areas over parasagittal regions of bilateral cerebral hemispheres, in keeping with multicystic encephalomalacia related to perinatal insults

The brain ultrasound at 22 days old of the second baby showed diffuse multiple cystic cavity formations of the cerebrum, predominantly in peritrigonal and parafalcial regions (Fig. 2). The corpus callosum was thin. At 6 months of age, the baby started to have dystonic movements and global delay. On examination, he had small head. His head circumference was 36 cm (<3rd centile) and hypertonia of four limbs. An electroencephalogram disclosed epileptiform discharge. Metabolic screening was normal. At 1 year of age, the head circumference was 38 cm (<3rd centile). His neck control was poor. He could not roll over from prone to supine. His four limbs were hypertonic and lower limbs were hyperreflexic. Magnetic resonance imaging of brain confirmed the diagnosis of extensive multicystic encephalomalacia. There were diffuse and severe white matter loss at bilateral cerebrum, severe and diffuse thinning of the corpus callosum and ex-vacuo dilatation of bilateral lateral ventricles (Fig. 3). The child developed epilepsy since 3 years old which was controlled with anti-epileptic medications. On last examination at 6 years of age, he still had severe global developmental delay. He could only vocalize but not yet speak. He could only sit and walk with support. A head computed tomography scan was performed for intractable seizure which revealed hypodense areas over parasagittal regions of bilateral cerebral hemispheres, in keeping with multicystic encephalomalacia (Fig. 4).

## Discussion

This report illustrated the postnatal neurological consequences in the survivors of monochorionic triplet pregnancy complicating by single intrauterine death in early gestation followed by fetofetal transfusion syndrome. Intrauterine fetal death in triplet pregnancies are not rare event. Dickey et al. [6] reported incidence of spontaneous reduction of triplet pregnancies was 53% (64/132, 95% CI, 44–61%) before 12 weeks of gestation. After 22 weeks of gestation, Kawaguchi et al. [5] reported intrauterine fetal death rates were 0.8% (12/1521), 2.8% (12/432) and 2.7% (4/150) for trichorionic triamniotic, dichorionic triamniotic and monochorionic triamniotic triplet pregnancies respectively. Few small case series and case reports are available in the literature describing the pregnancy outcomes [7, 8]. However, the majority of these studies had undetermined chorionicity and gestational age of fetal demise as well as unknown neurological follow up of the surviving triplets. Compared with triplet pregnancies, the profound adverse outcome of single twin demise in the second and third trimesters on the surviving co-twin has been well described [9–11]. A systematic review by Hillman et al. [9] found that among the surviving monochorionic twins, 34% had abnormal postnatal cranial imaging and 26% had neurodevelopmental impairment. The postulated mechanism was acute and transient transfusional event from the live fetus to the dead fetus through the placental vascular anastomoses leading to hypoxic-ischeamic injury of central nervous system because of hypoperfusion. Adverse effect on other body systems such as bowel, skin and limbs was also reported [12]. The anatomical distribution and pattern of cerebral hypoxic-ischaemic lesions would depend on time and duration of insult [13]. If hypotensive insult occurs before 28 weeks of gestation, multicystic encephalomalacia (MCE) or parenchymal haemorrhage is more likely to develop. MCE is diffuse lesion of the brain in which cerebral parenchyma is replaced by cysts of varying sizes in the perinatal period. It is believed to take around 4–6 months for the insult leading to permanent cortical brain tissue loss [14]. This type of brain lesion is associated with poor clinical consequences such as severe mental retardation, microcephaly, spastic tetraplegia, epilepsy and spastic hemiplegia [14–17]. On the other hand, the effect of single twin demise at first trimester is unclear. There are case reports about brain damage of surviving co-twins following single fetal death in monochorionic twin pregnancies in the first trimester. One case of fetal ventriculomegaly of MCDA (monochorionic diamniotic) co-twin after single fetal demise at 8–9 weeks [18]. One case of fetal MCE of MCDA co-twin after single fetal demise at 12 weeks [19]. Recent case series of 20 MCDA twin pregnancies with single fetal demise at 10–14 weeks, 12 cases had both fetal demise of both fetuses at the same time, 2 cases of twin reversed arterial perfusion sequence resulted in fetal demise of co-twins at second trimester [20]. The remaining six cases survived with normal neurological outcomes at least 1 year of follow-up [20]. Overall, the effect of co-twin demise is variable from no sequelae, different degree of multiorgan damages to subsequent demise, which may depend on the type of vascular anastomose in the shared placenta [21]. For triplet pregnancy, the effect of single demise in first trimester is unknown. The condition is likely under reported due to being missed on ultrasound scan or the dead co-triplet having been already absorbed by the time when dating scan is performed.

Besides, the other possible in-utero cause of multiorgan injury of the survivors in this case is the development of fetofetal transfusion syndrome. Fetofetal transfusion syndrome is a serious complication in monochorionic multiple pregnancies. It occurs in about 15% of monochorionic twin pregnancies [22], however the prevalence in triplet gestations is not known. The unbalanced blood flow via the placental arteriovenous anastomoses causes hypovolaemia/hypotension in one/two fetus (es) (donor) and hypervolaemia/hypertension in another fetus (es) (recipient) [23]. This haemodynamic instability can lead to multiple organ damage particularly the brain, and death. The low-flow and high flow injuries can result in vascular disruptive lesions [2]. In the fetal brain, hypoxic ischaemic injury to the white matter supplied by middle cerebral artery can cause extensive MCE Concurrently, haemorrhagic injury may occur in isolation or with concomitant ischemic lesions. Therefore, severe fetofetal transfusion syndrome exposes the donors and recipients to a higher risk of cerebral damage with wide spectrum of severity. In twin-twin transfusion syndrome, the prevalence of cerebral lesion was reported in 13–35% [24, 25] and the incidence of long-term neurological morbidity was reported in 10–25% [24]. However, there is lacking of this information in monochorionic triplet pregnancies.

Finally, the possible postnatal injury to brain and gastrointestinal tract cannot be excluded in this case. The prematurity and low birth weight are potential threats to the integrity of the brain which may worsen the long-term neurological outcomes of the survivors. However, it is difficult to ascribe the causes of final outcomes to solely complication of antenatal related single intrauterine death and/or fetofetal transfusion syndrome since the survivors were also exposed to postnatal sources of brain insults. Normal antenatal fetal brain imaging cannot exclude the in-utero brain insults because brain injuries can take months to evolve and allow cavitation formation, and only become evident on the radiological examination after birth. The first baby’s brain lesion was likely related to a result of prematurity, while the second baby’s MCE was likely due to severe intrauterine hypoxic ischaemic injury leading to cerebral necrosis. Antenatal administration of magnesium sulphate before early preterm delivery can help to alleviate the prematurity related long-term neurological impairment for these babies. However, this was not put into practise in our hospital at that time. Furthermore, the first baby suffered from jejunal atresia which might be related to discordant congenital anomaly. Congenital anomaly is commoner in multiple pregnancies than singleton pregnancies. The second baby suffered from gastric perforation which might be caused by prematurity or iatrogenic problem related to medical intervention after birth.

## Conclusions

We report the first case of brain and gastrointestinal injury in two surviving fetuses of monochorionic triplet pregnancy which may be associated with acute transfusion event related to single co-triplet death in the first trimester and chronic transfusion event related to fetofetal transfusion syndrome in early third trimester. Prematurity may have additional adverse effect on the long-term neurological outcome. One child suffered from MCE with cerebral palsy. Another child had periventricular leukomalacia and intracranial haemorrhage with normal neurodevelopment. Both had gastrointestinal tract damage requiring surgeries after birth. The present case highlights the high risk of multiple pregnancies, irrespective of whether they are twins or triplets. The major contributing factors were single fetal demise in early trimester, fetofetal transfusion syndrome and prematurity. Therefore, women should be counseled properly regarding the risk and offered fetal brain assessment of the surviving triplets using ultrasound and magnetic resonance imaging at least 4 weeks after the co-triplet demise to look for evidence of hypoxic-ischemic injury. They should be warned that even in the case of normal fetal brain imaging, cerebral injuries may appear later in pregnancy or in the neonatal period. Therefore, neonatal assessment and follow-up are important to assess for any neurodevelopmental problems.

## Data Availability

Not applicable.

## Abbreviations

MCDA: Monochorionic diamniotic
MCE: Multicystic encephalomalacia
